# Valproate-induced hyperammonemia - uncovering an underlying inherited metabolic disorder: a case report

**DOI:** 10.1186/s13256-018-1666-3

**Published:** 2018-05-17

**Authors:** Shaine Mehta, Sarrah Tayabali, Robin Lachmann

**Affiliations:** 1 General Practice, King’s College London, London, UK; 2 University College London Hospital, London, UK; 3 Metabolic Medicine, Charles Dent Metabolic Unit, National Hospital for Neurology and Neurosurgery, London, UK

**Keywords:** Sodium valproate, Encephalopathy, Liver disease, OTC

## Abstract

**Background:**

Sodium valproate is a commonly used anticonvulsant. It is widely recognized that valproate can cause hyperammonemia, particularly in people with underlying liver disease. Patients with urea cycle disorders are genetically predisposed to this adverse event and can develop severe hyperammonemia if given valproate.

This can occur even if liver functions tests and plasma concentration of valproate are normal, highlighting the importance of checking ammonia levels in any patient presenting with encephalopathy. Specific treatment for hyperammonemia must be implemented promptly.

**Case presentation:**

A 22-year-old white British man with a history of epilepsy post head trauma presented with subacute encephalopathy 4 weeks after the introduction of sodium valproate. His ammonia levels were not checked until 48 hours into his presentation and were found to be elevated. He initially responded to treatment of his hyperammonemia and the raised levels were attributed to sodium valproate. However, as his ammonia levels continued to rise, further investigation led to a diagnosis of ornithine transcarbamylase deficiency.

**Conclusions:**

Ornithine transcarbamylase deficiency is the most common of the urea cycle disorders. This case highlights both the importance of checking ammonia levels early and considering the diagnosis of this X-linked disorder in patients with raised ammonia, as these have implications both for the patient’s acute and further management, and for family screening.

## Background

Sodium valproate is a commonly used anticonvulsant. It is widely recognized that valproate can cause hyperammonemia, particularly in people with underlying liver disease. Valproate does this by modulating the levels of substrates of the urea cycle [1, 2]. This means that patients with urea cycle disorders are genetically predisposed to this adverse event and can develop severe hyperammonemia if given valproate.

Patients with valproate-induced hyperammonemia and encephalopathy may present with increased seizure frequency, lethargy, altered behavior or impaired consciousness. This can occur even if liver functions tests and plasma concentration of valproate are normal, highlighting the importance of checking ammonia levels in any patient presenting with encephalopathy.

Normally, valproate-induced hyperammonemia is transient and resolves if the drug is withdrawn. Not all patients with valproate-induced hyperammonemia are symptomatic. Withdrawal of the drug may not be warranted in asymptomatic cases, although close monitoring is advised. However, in patients with an underlying urea cycle disorder the effects of sodium valproate can be catastrophic, resulting in metabolic decompensation and progressive cerebral edema. Specific treatment for hyperammonemia must be implemented promptly.

Ornithine trancarbamylase (OTC) deficiency is the most common inherited cause of hyperammonemia. Most male hemizygotes will present in the neonatal period but in female heterozygotes and males with milder mutations presentation can be delayed. Presentation in adulthood is often missed as symptoms are nonspecific and hyperammonemia is seldom considered. Metabolic decompensation can be triggered by the introduction of drugs such as valproate, by high protein intake or by catabolic states such as intercurrent infection, fasting or during the post-partum period [2]. The concentration of ammonia may rise gradually and patients can present with subacute encephalopathy.

## Case presentation

A 22-year-old white British man with a background of epilepsy presented with a 2-week history of worsening nausea, vomiting, confusion, and aggressive behavior. On admission, he was encephalopathic and confused with a Glasgow Coma Scale of 14. In view of his deteriorating condition, he was admitted to the acute admissions unit for further investigation.

There was no history of significant illness in infancy or childhood. At the age of 18 while working as a lifeguard, he had a fall and hit his head, following this injury he had a grand mal seizure and was started on anticonvulsant medication. He had previously tried lamotrigine and levetiracetam but was unable to tolerate the non-neurological side effects. He did not report altered mental state on these medications. He had been started on sodium valproate 1 month prior to presentation.

Initial laboratory findings showed his alanine transaminase was elevated at 74 iU/L and his white cell count was 12 × 10^9^ /L. C-reactive protein and all other blood tests were within normal ranges. His ammonia level was measured 2 days later and was 120 umol/L (normal range: 12–47 umol/L). Despite weaning down and stopping sodium valproate, his ammonia level continued to rise, reaching 230 umol/L. At this time, specific therapy for his hyperammonemia was started.

Plasma amino acids showed a raised glutamine of 1160 umol/L (515–75), consistent with a chronic hyperammonemia and a low citrulline level of 13 umol/L (20–55), consistent with OTC deficiency. Urine organic acids showed a large peak of orotic acid, confirming the diagnosis.

Initially, it was thought that his encephalopathy was secondary to sepsis. It took 48 hours for the ammonia to be checked. The hyperammonemia was initially considered to be simply due to valproate therapy and the drug was stopped. When the ammonia continued to rise, an underlying metabolic disorder was considered.

To reduce the plasma ammonia levels, he was treated with sodium benzoate, sodium phenylbutrate, and L-arginine intravenously. He was also put on a zero protein diet and given intravenous 10% dextrose to prevent catabolism. Once his ammonia levels declined to below 100 umol/L, dietary protein was gradually reintroduced and sodium benzoate, sodium phenylbutrate, and L-arginine were continued orally. He commenced clobazam for his epilepsy.

He made a full recovery, with no neurological abnormality detected on examination at outpatient follow-up 2 months later. He cut down on his consumption of meat but otherwise follows a normal diet. Ammonia was normal at 35umol/L (12–47) at 2 months.

Following this presentation, he was referred to a specialist metabolic unit for further testing. He was found to have a genetic mutation associated with late presentation of OTC deficiency and continues to take reducing doses of medications for hyperammonemia with the aim to eventually stop them all together and get him back onto a normal diet in the long term.

He would be at risk of decompensation in the future if exposed to metabolic stresses such as prolonged fasting or severe gastroenteritis. He has been given an emergency dietary regime, consisting of high carbohydrate and low protein intake to follow in such circumstances. He has also been advised to obtain a MedicAlert bracelet.

As this is an X-linked condition, other family members have been recommended to attend for genetic screening.

## Discussion and conclusions

OTC is one of the five hepatic enzymes in the urea cycle which converts ammonia to urea, allowing renal excretion. Any disruption to this pathway can potentially lead to hyperammonemia and clinical encephalopathy [3]. More than 300 mutations causing OTC deficiency have been identified with most patients presenting in childhood, however, some mutations cause later presentation [4, 5]. Hyperammonemia in this latter group may be exacerbated by environmental factors such as the introduction of sodium valproate in this case. Males with milder forms may present with subtle behavioral changes and symptoms may be difficult to distinguish from psychiatric illness or drug abuse. Thus, urea cycle disorders must be considered at any age in a patient who presents with hyperammonemia.

Treatment of hyperammonemia is similar to the treatment of hepatic encephalopathy and should be started immediately. The aim is to reduce ammonia production by stopping catabolism. Ammonia is produced from the breakdown of dietary proteins and in catabolic states, endogenous protein. Treatment aims to stop protein intake and stop catabolism by providing calories as carbohydrates using intravenous glucose and lipids. Ammonia can be removed, using the alternative pathway scavengers used in this case (sodium benzoate and sodium phenylbutyrate) [6]. In proximal urea cycle disorder, L-arginine is also used as it stimulates protein synthesis. In severe hyperammonemia hemodialysis or hemofiltration may be required. Dietary protein is normally reintroduced after 48 hours to avoid essential amino acid deficiency, as this would increase endogenous protein catabolism. Reintroduction of protein requires specialist dietetic guidance. Early discussion with a metabolic team is important and the patient is likely to need referral to a specialist center after the acute episode.

With any inherited genetic disorder there are implications for family screening. Other urea cycle disorders have an autosomal recessive pattern of inheritance, however OTC is X-linked, which has specific implications. Affected men are advised that while their sons will be unaffected, their daughters will become carriers of the gene. The daughters should be advised that they have a 50% chance of passing the gene onto their offspring, and that both female offspring and male offspring may present with late-onset disease. In this case, as the mutation resulted in late-presenting disease, affected male offspring are unlikely to present with severe disease in the neonatal period. While female carriers are less likely to be affected, skewed X-inactivation in the liver can lead to the development of hyperammonemia.

In the long term, patients who have a hyperammonemic episode need advice about the possible need for dietary modification, avoiding dehydration, fasting and other triggering events, and the use of emergency regimes at times of metabolic stress. Not all of these patients may need to take long-term nitrogen scavengers, however, this may be considered later in life as the disease can get more brittle with age. Decisions also need to be made regarding the care of asymptomatic individuals with OTC mutations. Periodic follow-up in a specialist center is recommended.

Key learning points:

- Ammonia levels should be checked in all encephalopathic patients.
- Remember that sodium valproate can cause hyperammonemia.
- Urea cycle disorders may present in adulthood.
- Prompt diagnosis can facilitate rapid treatment, with appropriate input from a metabolic consultant.
- The diagnosis of a genetically inherited disease has implications for other family members. Adequate counseling of family members is essential.

## Abbreviations

OTC: Ornithine trancarbamylase (OTC)
